# Changes in fecal microbiota after therapeutic exposure to amoxicillin-clavulanic acid in veal calves receiving multiple antibiotics

**DOI:** 10.1128/spectrum.01316-25

**Published:** 2025-11-10

**Authors:** Morgane Moustaghfir, Thibault Destanque, Pauline François, Pierre Châtre, Vanessa Louzier, Abdessalem Hammed, Karine Hauray, Jean-Yves Madec, Marisa Haenni, Caroline Prouillac, Agnese Lupo

**Affiliations:** 1 ANSES — Université de Lyon, Unité Antibiorésistance et Virulence Bactériennes 133614, Lyon, France; 2 VetAgro Sup Campus Vétérinaire de Lyon, Université de Lyon 133614, Lyon, France; 3 Clinique Vétérinaire du Clair Matin, Bourg-en-Bresse, France; University of Georgia College of Veterinary Medicine, Athens, Georgia, USA

**Keywords:** amoxicillin-clavulanic acid, veal calves, microbiota, 16S V1-V9 sequencing, *Escherichia coli*, ESC-R, *bla*_CTX-M_, intI1, Lachnospiraceae, ST2325

## Abstract

Amoxicillin-clavulanic acid (AMC) is an important antibiotic in human and veterinary medicine. However, it can select antibiotic-resistant bacteria in the gut microbiota and alter its composition. In calves, AMC can be used to treat complicated infections. The impact of AMC on the calves’ gut is unknown; thus, we analyzed the effect of a five-day AMC treatment in veal calves suffering from omphalitis. AMC-treated calves (*n* = 15) and a control untreated group (*n* = 15) were enrolled from two farms. Stools were collected before AMC administration (D0), one day (D6), one month (D35), and two months (D55) after AMC withdrawal. The effect of AMC treatment on gut microbiota composition and resistance gene selection was analyzed by full-length 16S rRNA operon sequencing and qPCR, respectively. Selection of Extended-Spectrum Cephalosporin-Resistant (ESC-R) *Escherichia coli* in calf’s fecal samples and dissemination in the farms’ environment were analyzed by cultivation and genome sequencing of isolates. After AMC treatment, alpha-diversity decreased in AMC-treated calves along with a decrease of the relative abundance of bacterial families beneficial for host health (Lachnospiraceae) and an increase of Pseudomonadota, grouping opportunistic pathogens. ESC-R *E. coli* carriage dynamics were different between calves sampled from each farm and seemed independent from AMC treatment. Besides, calves received other antibiotics that could have an impact on resistance selection. Certain clones of ESC-R *E. coli* demonstrated a widespread dissemination in the farm, both in calves hosted in distant zones and in their environment. To decrease the antibiotic resistance burden, it is essential to use antibiotics judiciously, alongside improving hygienic practices in farms.

**IMPORTANCE:**

Antibiotic therapies can select resistant bacteria in the gut of treated hosts and deplete bacteria that are beneficial to the host health. Antibiotic-resistant bacteria selected in the gut of food-producing animals, like veal calves, are excreted and can then disseminate among animals, to the environment (through manure or water contamination) and to farmers who may further disseminate these organisms to other people in contact. Antibiotic resistance genes can disseminate among clones present in the gut of both animal and human hosts by horizontal gene transfer. Studying the impact of antibiotic therapies on the gut microbiota has One Health relevance. Thus, we aimed to (i) analyze the impact of AMC treatment on the selection of resistant bacteria in the calf gut and its composition and (ii) analyze the dissemination resistance in farms in order to advise on potential strategies to counteract further spread of these microorganisms.

## INTRODUCTION

The gut microbiota has a mutualistic role with its host, contributing to digestive functions, protecting against pathogens, and regulating metabolic and immune systems (1). Antibiotic therapies, independently from their administration route, are major disruptors of gut microbiota composition, causing the decrease of bacterial diversity (2). In particular, antibiotics decrease the relative abundance of species belonging to the beneficial Bacillota phylum, favoring the expansion of other phyla, like Pseudomonadota, that includes opportunistic pathogens and carriers of antibiotic resistance genes (3). These effects have been observed in both humans and animals, including calves (4, 5).

The co-administration of amoxicillin and clavulanic acid (AMC) is active against several bacterial species, including those producing class A beta-lactamases (6). In humans, AMC is used to treat diverse infections affecting the urinary tract, the upper and lower respiratory tract, or the skin and soft tissue (7). In calves, AMC can be used to treat diarrhea and complicated omphalitis (8, 9). Due to its capacity to effectively treat a wide range of bacterial infections, AMC is an important antibiotic of the therapeutic arsenal in both humans and veterinary medicine. However, similar to other antibiotics, it can cause perturbation of the gut microbiota, potentially resulting in diarrhea emergence (10) and selecting resistant bacteria (10, 11). The European Medicine Agency (EMA) has categorized AMC as a class C antibiotic (“caution”) that can be used to treat animals since there are alternatives in human medicine but should nevertheless be used only when the first line options (class D, “prudence”) like amoxicillin without beta-lactamase inhibitor are not clinically effective. The intent is to spare antibiotics necessary to treat complicated infections or able to overcome certain resistance mechanisms (12).

France is a major contributor in the sector of veal calves farming (13), often carried out in intensive conditions. During the pre-weaning period, when the gut microbiota is more vulnerable to infective agents and to changes (14), veal calves are subjected to stressful management, leaving the farm of birth and being transported in trucks to fattening centers. During transport, they are under stress due to overcrowding and loud noises, but they also experience privation of food and water that can result in different degrees of dehydration (15). Thus, calves often receive antibiotics upon arrival in the fattening farm (16), which can affect gut microbiota and select antibiotic-resistant bacteria. In this context, extended-spectrum cephalosporin-resistant *Escherichia coli* (ESC-R *E. coli*) represents a critical issue (17). In the gut, ESC-R *E. coli* can disseminate by clonal expansion or by horizontal transfer of plasmids carrying the genetic determinants of this resistance (17). ESC-R *E. coli* can be excreted from the gut and then disseminated among animals, to farm facilities, to the environment, and to humans (18, 19). Most commonly, ESC-R *E. coli* carry genes of the *bla*_CTX-M_ type encoding extended-spectrum beta-lactamases (ESBL) (19, 20) or mutations in the promoter or attenuator regions of the intrinsic *ampC* gene (21, 22).

Previous studies have analyzed the impact of several antibiotic therapies in calf’s gut (23). They demonstrated that a three-day trimethoprim-sulfamethazole therapy in calves delayed gut microbiota maturation (24), or that a five-day oxytetracycline therapy correlated with the decrease of Bacillota and selection of tetracycline resistance genes in veal calves (25). Previously, we have investigated the effect of amoxicillin therapy on the gut microbiota of veal calves, highlighting a decrease of bacterial diversity and an increase of the copy number of antibiotic resistance genes (26). Furthermore, amoxicillin therapy co-selected genes conferring resistance to other antibiotic classes (*tetA*, *strA,* and *strB*) and class one integrons, indicators of multidrug resistance (26). Besides the clinical importance of AMC, studies analyzing the impact of AMC therapy on gut microbiota of calves are unreported.

This investigation aimed to analyze the effect of a five-day AMC therapy by intramuscular injection on the gut composition and antimicrobial resistances of calves suffering from omphalitis. Furthermore, the study aimed to investigate the dissemination of ESC-R *E. coli* among calves and in the farm environment during and after AMC therapy.

## MATERIALS AND METHODS

### Calves’ cohort and fecal samples collection

According to Directive 2010/63/UE, all procedures conducted in this study were approved by the Institutional Animal Care and Use Committee of VetAgro Sup (MENESR 2023062923119337). Calves were enrolled in two intensive production farms (A and B) located in France, in the Auvergne-Rhône-Alpes region. Fifteen calves (10 from farm A and 20 from farm B) suffering from omphalitis received a five-day AMC treatment (7 mg/kg, Synulox, Zoetis, France) by intramuscular injection every 10 (±2) h. Fifteen other calves, not receiving AMC, were enrolled as control of the same age as the treated ones and originating from the same department (Table S1). The 30 veal calves belonged to four different breeds (Prim’ Holstein, Crossed breed, Belgian Blue, Swiss Brown) were male, aged between 16 and 38 days, weighed approximately 50 kg (Table S1).

The first stools were collected before AMC treatment (day 0, D0), within 48 h after the arrival to the farm, one day (day 6, D6), one month (day 35, D35), and two months (day 55, D55) after AMC withdrawal. Stools were taken from the calves’ rectum, placed in single-use sterile containers, and transported in refrigerated boxes to the ANSES-Lyon laboratory for bacteriological and genomic analysis.

**Fig 1:**
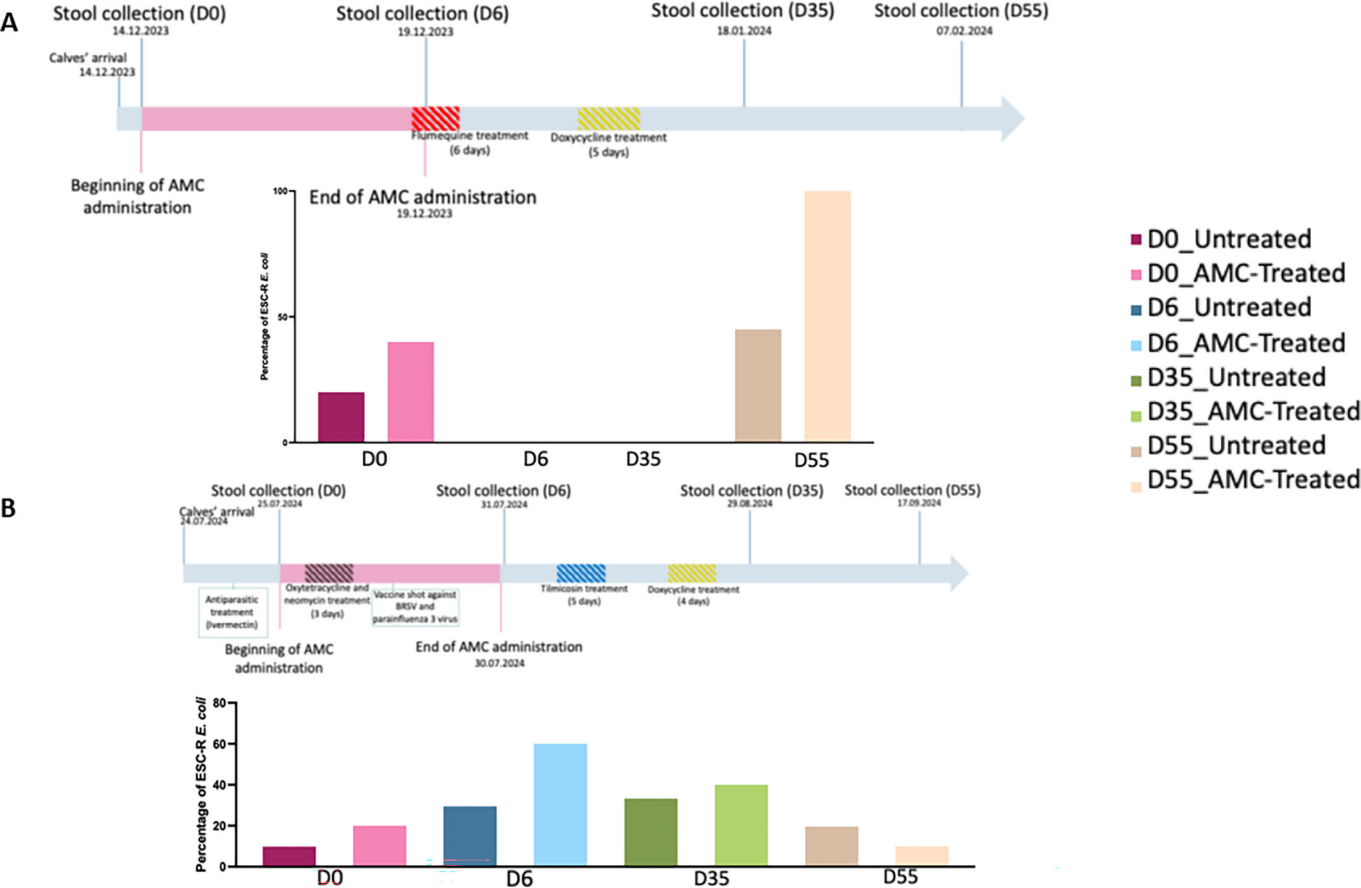
Timeframe of stool sampling, treatments, and events recorded during the study period in farm A (**A**) and in farm B (**B**) and occurrence of extended-spectrum cephalosporin-resistant (ESC-R) *Escherichia coli* isolates in calves. AMC-treated calves are those that received amoxicillin-clavulanic acid (AMC) treatment and untreated calves are those not receiving AMC.

At their arrival and until D6, calves were housed in individual boxes that allowed contact only with a neighboring calf. Afterwards, calves circulated in a space containing ten individuals, on average (Fig. S1). Additionally, to AMC treatment, all calves from farm A received orally, through milk replacer, at D4, flumequine (12 mg/kg) for intestinal problems, and doxycycline (10 mg/kg) at D12, to treat respiratory conditions, for six and five days, respectively (Fig. 1A). In farm B, at D0, all calves received an antiparasitic treatment (Ivermectin, 0.2 mg/kg) and an intranasal vaccine shot against bovine respiratory syncytial virus and parainfluenza 3 virus. At D1 and for three days, all calves of farm B received oxytetracycline (10 mg/kg) and neomycin (40 mg/kg) orally, through milk replacer, for intestinal disorders. At D6, they also received tilmicosin (10 mg/kg), and at D16, doxycycline (10 mg/kg), for five and four days, respectively, through milk replacer to treat respiratory conditions (Fig. 1B).

### 16S rRNA (V1–V9) sequencing

DNA was extracted from frozen stools (250 mg) using the DNA mini Stool Kit (Macherey Nagel, Hoerdt, France). DNA from a predefined bacterial community (250 µL) used as a microbiota quality control was also extracted (TruMatrix Technology, ZymoBIOMICS, Freiburg, Germany). DNA concentration and purity were determined by absorbance (NanoDrop, Ozyme, Saint-Cyr-l'Ecole, France) and by fluorometric assay using the dsDNA Broad Range Kit (Qubit 4, Thermo Fisher Scientific, Illkirch, France). Community DNA (10 ng) together with the LongAmp Hot Start Taq 2X Master Mix (New England Biolabs, Evry, France) and 27F and 1492R primers were used for the amplification of the V1–V9 region of the 16S rRNA gene. Sequencing was performed using the MinIon instrument (Nanopore Oxford Technology) (27). Sequencing reads were processed using epi2me/wf-metagenomics (v.2.10.0) (Supplementary material 1).

### ESC-R *Escherichia coli* analysis

To investigate the dissemination of ESC-R *E. coli* within the farms, stools were collected from 15 (farm A) and 41 (farm B) additional calves not treated with AMC (Table S1), accounting for 10% of calves hosted in each farm. Additionally, environmental samples (*n* = 40 in each farm) were collected at each sampling time, at the same location, and included drinking troughs, feeders, calf nipples for nursing bottles, and door handles (eSwab, Biomérieux, Marcy l’Etoile, France). Stools and eSwab were streaked without enrichment step on selective ESBL and mSuperCARBA medium (CHROMagar, Saint-Denis, France) for the detection of *E. coli* resistant to extended-spectrum cephalosporins and carbapenems. After 24 h incubation at 37°C, three presumptive *E. coli* isolates (red/pink colonies) were streaked on MacConkey agar for purification (BioRad, Marnes-la-Coquette, France). The assignment of *E. coli* isolates to the four major phylogenetic groups (A, B1, B2, and D) was performed as described by Doumith et al. depending on the presence/absence of the *chuA*, *yjaA*, TSPE4.C2, and g*adA* genes (28). The multiple-locus variable-number tandem-repeats (MLVA) method described by Camelena et al. (29) (a multiplex PCR containing seven couples of primers) was used to unveil genetic similarities among *E. coli* isolates. Obtained amplicons were separated by electrophoresis and banding patterns evaluated visually. Isolates presenting a unique profile from a unique calf and per sample time (*n* = 104) were further characterized.

Minimum Inhibitory Concentration (MIC) of 14 antibiotics was evaluated by microdilution using Sensititre CMV3AGNF plates (Thermo Fisher Scientific) following manufacturer’s instructions and *E. coli* ATCC 25922 strain as quality control. Susceptibilities were interpreted according to the Antibiogram Committee of the French Society for Microbiology (CA-SFM; https://www.sfm-microbiologie.org/2023/06/15/casfm-veterinaire-2023/ and https://www.sfm-microbiologie.org/wp-content/uploads/2020/07/CASFM_2013.pdf for veterinary-related antibiotics and for human-related antibiotics, respectively).

DNA of all non-duplicate isolates was extracted (NucleoSpin microbial DNA, Macherey Nagel), and short-read sequencing was outsourced (Illumina NovaSeq 6000 technology, Eurofins Genomics, Ebersberg, Germany). Details of bioinformatic analyses on *E. coli* genomes are provided in Supplementary material 1.

### Quantification of selected genes conferring antibiotic resistance

Quantification of genes potentially selected during AMC therapy (*bla*_TEM_), detected among ESC-R *E. coli* isolates (*bla*_CTX-M-1-9-groups_, *strB*, *qnrS/qnrB1, sul1/sul2, tetA*), or indicators of multidrug resistance (*intI1*) was determined according to the number of copies for each gene and for the 16S rRNA encoding gene using hydrolytic probes (30). The ratio of the number of copies of each gene on the 16S rRNA gene was calculated.

### Statistical analyses

A Wilcoxon test was performed to estimate differences in alpha-diversity between AMC-treated and untreated veal calves and between the different time samples. For the beta-diversity, a Bray-Curtis dissimilarity analysis was represented on a Non-Metric Multi-Dimensional Scaling (NMDS) plot (Supplementary material 1). For the quantification of antibiotic resistance genes, a Wilcoxon test was performed to assess the difference in number of copies of quantified genes between the AMC-treated and untreated calves at each sampling time.

## RESULTS

### Calves’ gut microbiota analysis after AMC exposure

**Fig 2:**
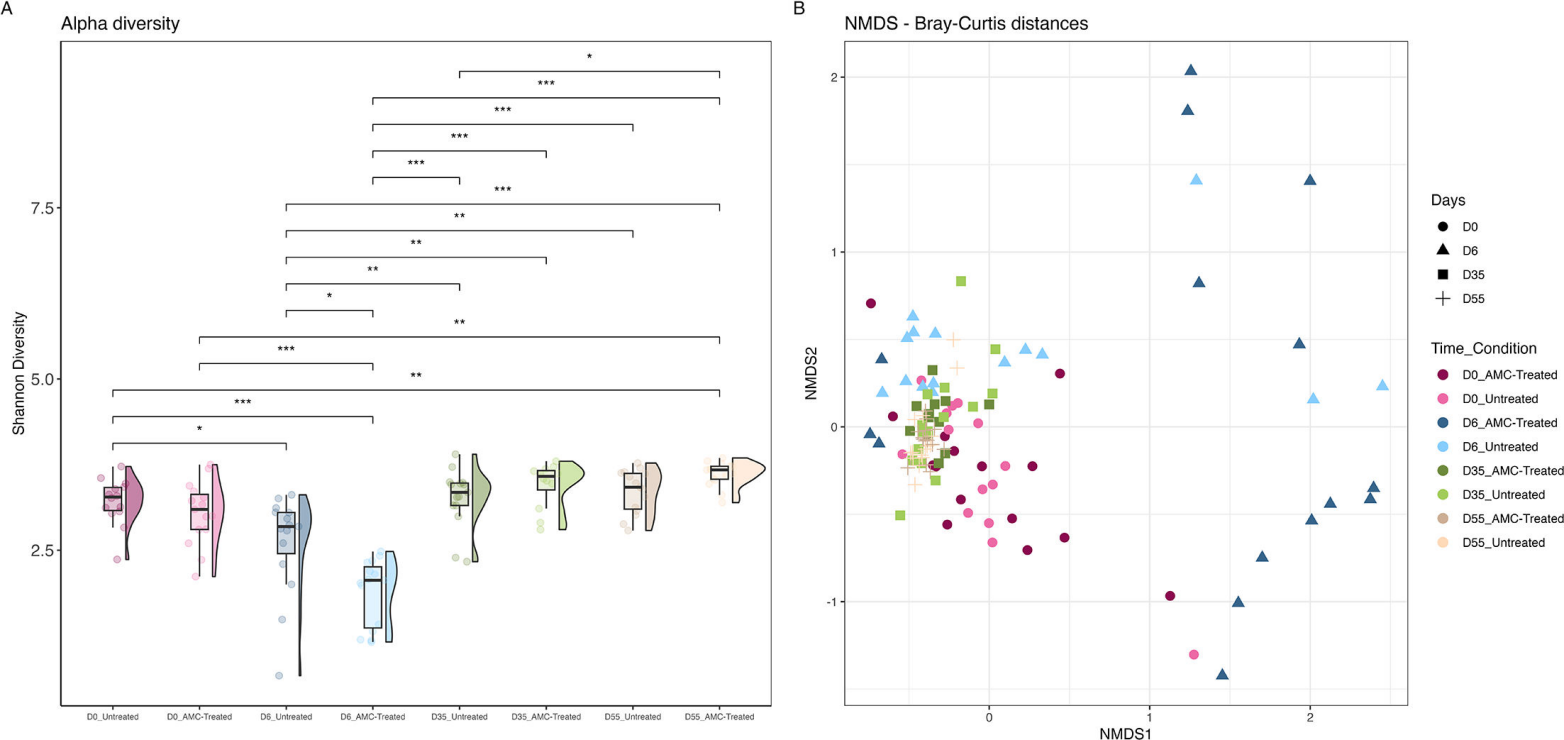
(**A**) Alpha-diversity (Shannon index) of the gut microbiota based on the sequencing of the V1–V9 region of the 16S encoding operon (Oxford Nanopore Technologies, MinION). Stools were analyzed from 15 AMC treated and 15 untreated calves before AMC administration (D0), one day after AMC withdrawal (D6), one month (D35), and two months (D55) after AMC withdrawal. Only significant comparisons are shown (*: *P* ≤ 0.01; **: *P* ≤ 0.001; ***P ≤ 0.0001, Wilcoxon test). (**B**) Bacterial beta-diversity was calculated using Bray-Curtis dissimilarity distances (stress value = 0.15, *R* = 116). PERMANOVA analysis, using pairwise comparisons on distance matrix, showed significant bacterial differences across the time samples (*P* = 0.001, *R*^2^ = 0.229) and between the two conditions (*P* = 0.017, *R*^2^ = 0.019).

Upon arrival at the farms, both AMC-treated (*n* = 15; 5 from farm A, 10 from farm B) and untreated calves (*n* = 15; 5 from farm A, 10 from farm B) exhibited similar levels of alpha-diversity in their gut microbiota (Fig. 2A). Following AMC treatment (D6), a decrease (*P* ≤ 0.0001) of alpha-diversity was observed in AMC-treated calves compared to D0 and to untreated calves. Alpha-diversity decreased also in untreated calves at D6 (*P* ≤ 0.01). In both calves’ groups, alpha-diversity increased already one month after AMC withdrawal (D35; AMC-treated, *P* ≤ 0.0001; Untreated, *P* ≤ 0.001). Two months post-AMC withdrawal (D55), both groups of calves exhibited alpha-diversity greater than that estimated at D0 (*P* ≤ 0.001) (Fig. 2A).

The composition of calves’ microbiota significantly changed over time and between the two groups of calves (Fig. 2B). The Bray-Curtis analysis displayed that sampling time and treatment accounted for 18% and 2% of the observed diversity, respectively. At D0, AMC-treated and untreated calves displayed similar gut composition, similar to what was observed at D35 and D55. At D6, 12 out of 15 AMC-treated calves presented a distinct composition compared to gut microbiota sampled at D0, D35, and D55 (Fig. 2B).

**Fig 3:**
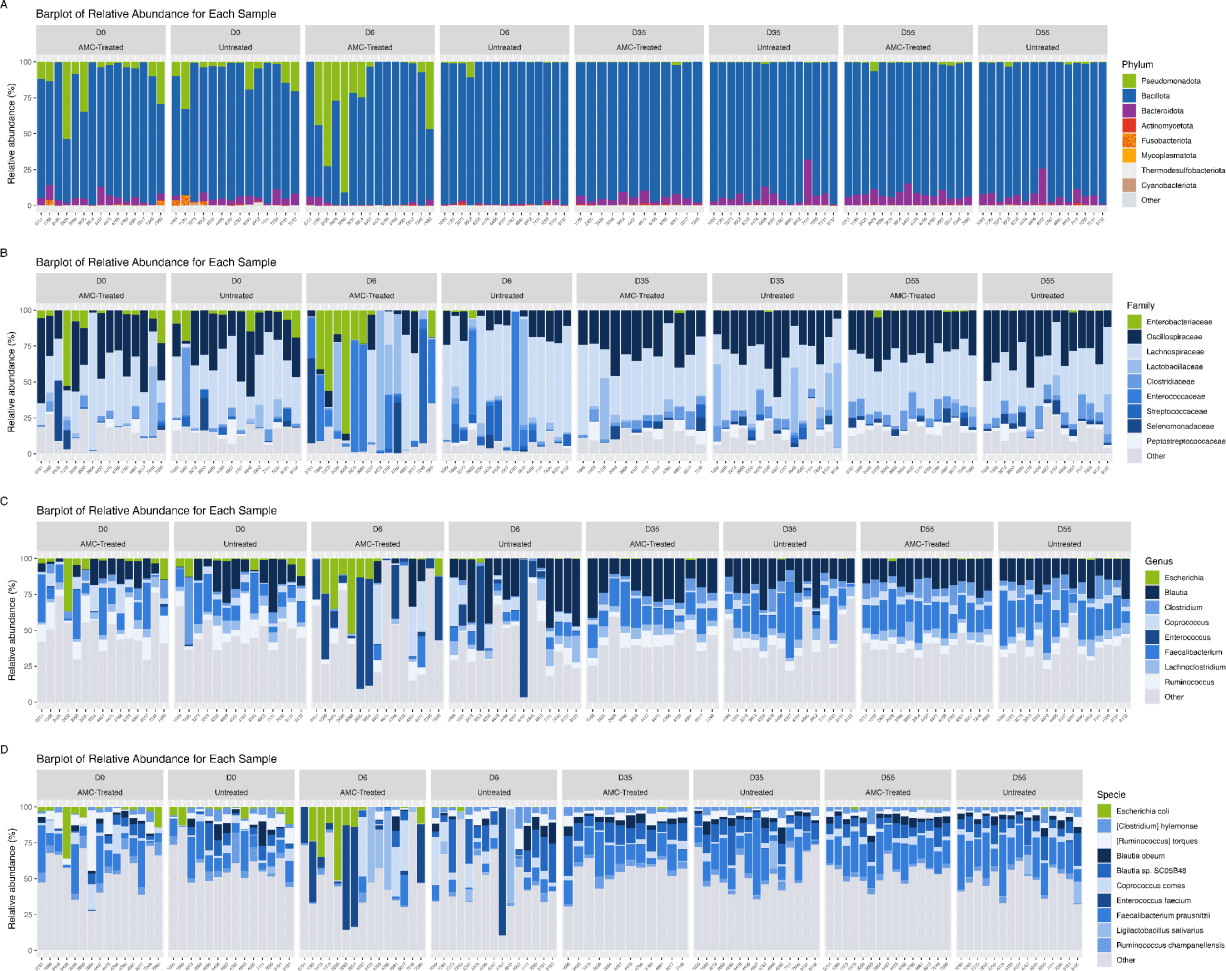
Relative abundance of the fifteen most abundant phyla (**A**), families (**B**), genera (**C**), and species (**D**).

Such dissimilarity was corroborated by the relative abundance across various taxonomic levels (Fig. 3). At the phylum level, no significant differences in relative abundance were observed between AMC-treated and untreated calves at D0, D35, and D55, during which the microbiota of both groups was dominated by Bacillota (Fig. 3A). At the family level, Lachnospiraceae and Oscillospiraceae were the most abundant (Fig. 3B; Table S2), with the genera *Faecalibacterium*, *Blautia*, *Coprococcus*, and *Ruminococcus* being more abundant (Fig. 3C; Table S2).

Pseudomonadota dominated the microbiota of AMC-treated calves at D6 (Fig. 3A). Enterococcaceae and Enterobacteriaceae were the most abundant families, the latter undergoing a four-fold increase compared to D0 and being 23-fold more abundant in AMC-treated calves compared to untreated ones (Fig. 3B; Table S2). Moreover, families regrouping opportunistic pathogens, such as Yersiniaceae and Morganellaceae, were more abundant in AMC-treated calves (Table S2).

Consistent with the families’ relative abundance, the genera *Escherichia* and *Shigella* underwent a five-fold increase between D0 and D6 when they were 37- and 44-fold more abundant in AMC-treated calves (Table S2). The species *Escherichia coli* underwent a 29-fold increase at D6, and it was 38-fold more abundant in AMC-treated calves (Fig. 3D; Table S2).

Similarly, *Proteus* was 29-fold more abundant in the AMC-treated group at D6. No genus within the Enterococcaceae family presented differential abundance between the two groups. This family underwent a ten- and six-fold increase from D0 to D6 in AMC-treated and untreated calves, respectively (Table S2). The genus *Blautia* and species belonging to this genus (*Blautia* sp. SC05B48) were less abundant in AMC-treated calves at D6 (Fig. 3C and D; Table S2).

At D6, in the microbiota of untreated calves, Acidaminococcaceae, Eubactericeae, Lachnospiraceae, Peptostreptococcaceae, Streptococcaceae, and Veillonellaceae exhibited significantly greater abundance compared to AMC-treated calves (Table S2). In agreement with the family analysis, *Eubacterium* and *Mogibacterium* genera were 11- and 100-fold more abundant in untreated calves (Table S2).

### Occurrence of ESC-R *Escherichia coli* isolates and resistance determinants

AMC-treated and untreated calves were screened for the presence of ESC-R and carbapenem-resistant *E. coli* isolates. To analyze the dissemination of these organisms in the farm and achieve a 10% representation of the resident population, additional calves were screened. In total, 25 and 61 calves were screened in farm A and B, respectively (Table S1). While no carbapenem-resistant *E. coli* was detected, few calves were colonized at the arrival to the farms with ESC-R *E. coli* (Table S1).

In farm A, only three calves were colonized with ESC-R *E. coli* at their arrival (D0), of which two received AMC. No ESC-R *E. coli* was detected at D6 and D35, whereas at D55, all AMC-treated calves (5/5) and 25% of untreated calves (5/20) were positive (Fig. 1A). Isolates colonizing calves at D0 differed from those retrieved at D55 (Table S1). In farm B, at D0, 9.8% (6/61) calves were ESC-R *E. coli* carrier, with one of these six calves included in the AMC-treated group. At D6, 50% (5/10) of AMC-treated and 29.4% (15/51) of untreated calves were ESC-R *E. coli* positive (in total 32.8%, 20/61). At D35, 30% (3/10) of AMC-treated and 35.3% (18/51) of untreated calves were carriers. At D55, only 1/10 of the AMC-treated group was positive, whereas 23.5% (16/51) of untreated calves were colonized with ESC-R *E. coli* (Fig. 1B; Table S1).

Overall, ESC-R *E. coli* isolates collected from veal calves were resistant to ceftiofur (91.3%; *n* = 95/104; MIC_50/90_: >8 mg/L, Table S3) with 90 isolates carrying gene from the *bla*_CTX-M-1_ group (*bla*_CTX-M-1_ *n* = 69, 76.7%; *bla*_CTX-M-15_ *n* = 6, 6.7%; *bla*_CTX-M-55_ *n* = 14, 15.6%; *bla*_CTX-M-32_ *n* = 1,1.1%) belonging to diverse STs (Tables S1 and S3). In particular, isolates carrying the *bla*_CTX-M-1_ gene belonged to ST10, ST34, ST48, ST58, ST362, ST744, ST2325, ST11905, isolates carrying *bla*_CTX-M-15_ belonged to ST46, ST1722, ST4681, ST4981, whereas isolates carrying *bla*_CTX-M-32_ and *bla*_CTX-M-55_ belonged to ST4471 and ST744, respectively. Five ESC-R *E. coli* carried a *bla*_CTX-M-14_ gene variant belonging to the *bla*_CTX-M-9_ group (Table S3). *In silico* analysis suggested that *bla*_CTX-M-1_ gene was located on a plasmid (*n* = 37) and on the chromosome (*n* = 15). For 17 isolates, the location remained undetermined. The location of all *bla*_CTX-M-55_ was predicted on a plasmid, whereas all *bla*_CTX-M-14_ were predicted on the chromosome, similar to *bla*_CTX-M-15_ that was predicted on the chromosome for five isolates out of six. Four isolates were cefoxitin-resistant (*n* = 4/104; MIC_50/90_: 4 mg/L) due to mutations in the *ampC* promoter region and belonged to ST88 (Table S3). Four isolates had reduced susceptibility to ceftriaxone (MIC: 2 mg/L), carried a *bla*_SHV-12_, and belonged to ST10. The location of *bla*_SHV-12_ was only determined for two isolates and was predicted on the chromosome. Nineteen isolates were AMC-resistant (MIC >16/8 mg/L) and carried a *bla*_TEM_ gene (Table S3). Occurrence of tetracycline and streptomycin resistance was common (*n* = 104 and 100, respectively), with genes *tet(A)* (*n* = 73) and *strA/strB* (*n* = 83) as the most prevalent. Ciprofloxacin resistance was less frequent (*n* = 27/104; MIC_50_: ≤0.015 mg/L, MIC_90_: >4 mg/L) associated with mutations in the quinolone resistance-determinant region (Table S3).

### Genetic diversity of ESC-R *Escherichia coli* isolates

**Fig 4:**
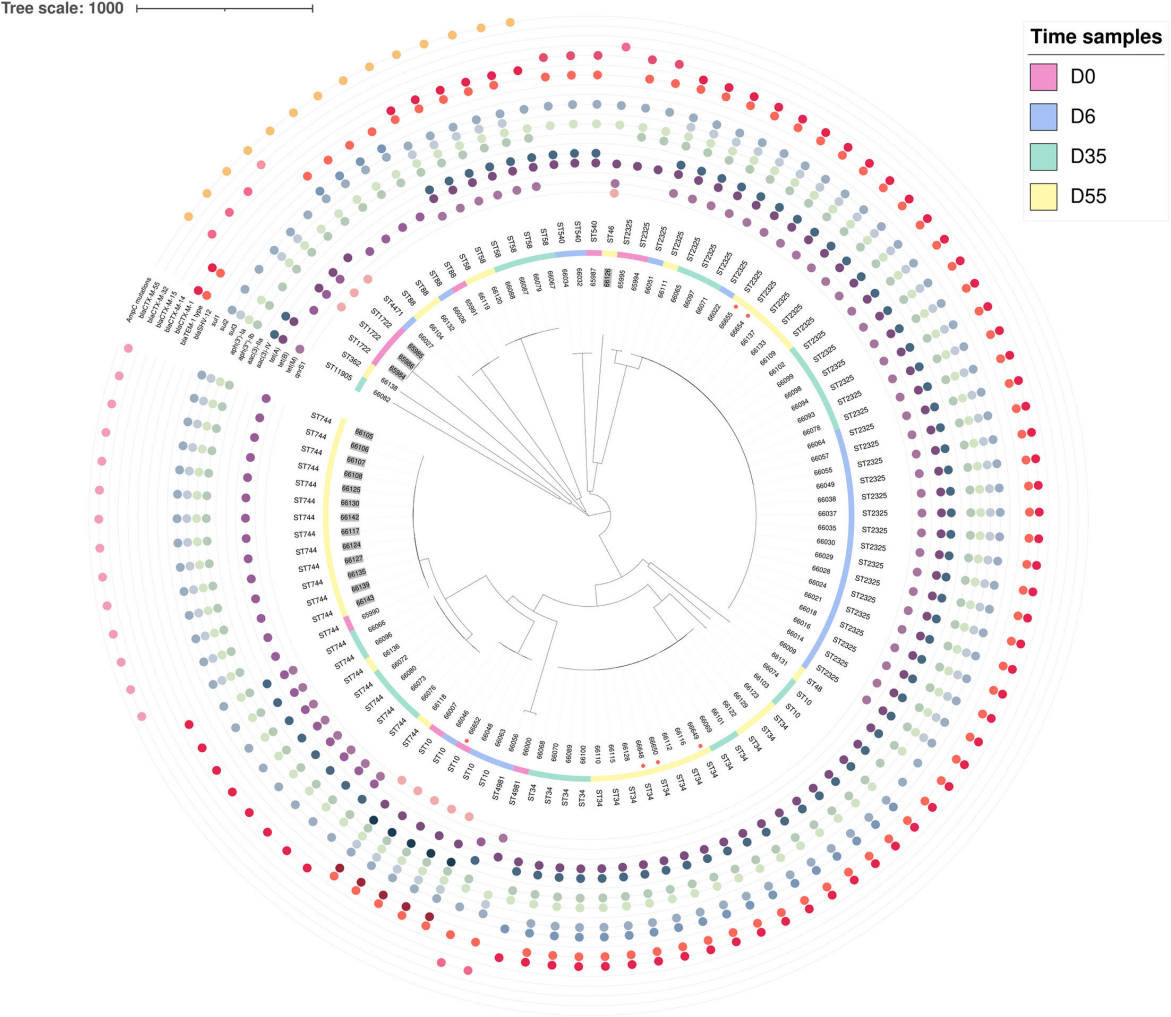
Representation (iTOL v7) of cgMLST-based tree of 104 ESC-R *E. coli* isolates from AMC-treated and untreated veal calves and from the environment of the fattening centers. Isolates from farm A are gray shadowed. Orange dots indicate isolates from the farms’ environment.

Upon arrival at farm A, three calves carried an ST1722 ESC-R *E. coli*, not detected on the following samplings (Fig. 4; Table S1). At D6 and D35, no ESC-R *E. coli* was detected. At D55, ESC-R *E. coli* ST744 sharing the same antibiotic resistance genes and plasmid content (IncFIB and IncQ1) disseminated among 13 different calves (SNPs, min: 0; max: 4) (Fig. 4; Fig. S2A).

In farm B, ESC-R *E. coli* isolates (*n* = 6) detected in calves at their arrival were more diverse, belonging to ST744, ST2325, ST10, ST540, ST88, ST4981. One ESC-R *E. coli* ST10 was recovered from a feeder (Fig. 4). The ST2325 ESC-R *E. coli* that disseminated at D0 diverged from isolates collected at subsequent samplings, whereas those detected in calves at D55 exhibited high genomic similarities with isolates from the environment (1–4 SNPs) and originated from calves situated in farm zones 1 and 8 (Fig. S1 and S2B; Table S4). Similarly, ST744 ESC-R *E. coli* detected at D0 diverged from the eight isolates collected at D35 and D55 in the farm zone 4 (Fig. S1 and S2A; Table S4). The ST10 ESC-R *E. coli* was recovered from a calf and a feeder at D0 and was found in three calves inhabiting zone 3 at D55. At D35, two ST10 ESC-R *E. coli* differing from those found at D6 emerged (Fig. S1 and S2C; Table S4).

The ST34 (*n* = 17) and ST58 (*n* = 6) ESC-R *E. coli* emerged at D35 and persisted at D55. The ST34 isolates differed by a maximum of 12 SNPs. Isolates 66070 and 66115 were indistinguishable and were found in a unique calf at different samplings, demonstrating the ability of the clone to persist in the calves’ gut at least for two weeks. Isolates present on a calf nipple were close to isolates from calves’ feces. The ST34 isolates colonized multiple calves in zones 1 and 8. Despite the core-genome proximity of ST34 isolates, three had different antibiotic resistance genes or plasmid content, suggesting recent acquisition or loss of these determinants (Fig. S2D). The ST58 ESC-R *E. coli* emerged at D35 in three calves of farm B and was found in one calf at D55. Calves were hosted in three adjacent zones (1–3). Two isolates (66087 and 66088) were collected at D35 from a unique calf but differed by 8 SNPs. Similarly, two isolates collected at D55 (66119 and 66120) diverged by 8 SNPs and originated from a unique calf (Fig. S1 and S2E).

### Quantification of genes conferring antimicrobial resistance

Upon arrival at the farms (D0), the 15 AMC-treated and 15 untreated control calves resulted positive for the presence of *sul1, sul2, intI1, strB*, *bla*_TEM_, and *tet(A*) genes. A variable number of calves was positive at D0 for *qnrS* (*n* = 7), *qnrB1* (*n* = 2), and *bla*_CTX-M-1_ (*n* = 3) and at the other sampling points (Table S5).

**Fig 5:**
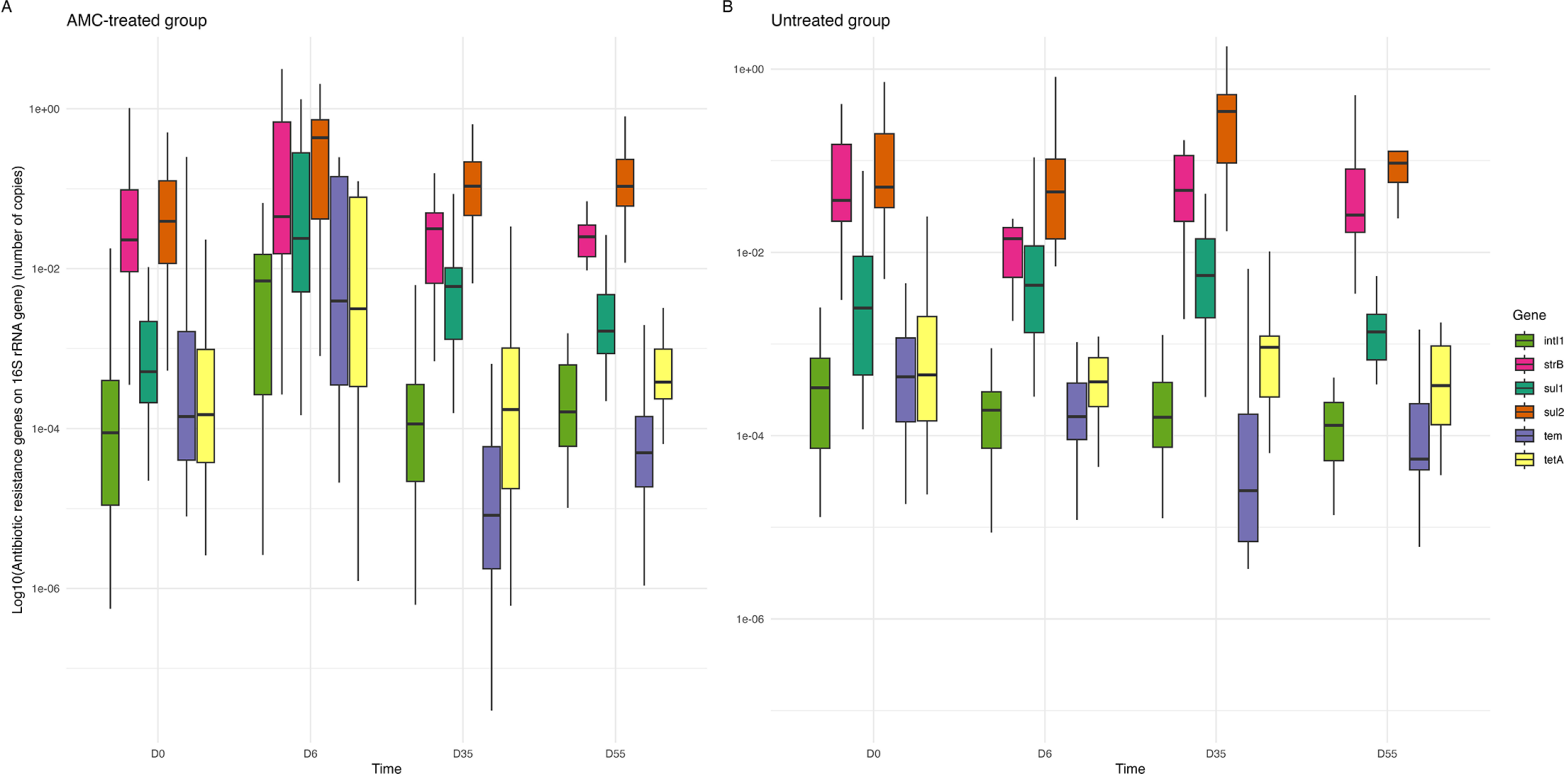
Relative quantification of 10 selected antibiotic resistance genes between AMC-treated (**A**) and untreated (**B**). A Wilcoxon test was performed for each gene, between each condition at the different time samples.

In untreated calves, the relative quantification of most genes (*bla*_TEM_, *strB*, *sul1*, *sul2*, *tet(A),* and *intI1*) remained constant over the sampling period (Fig. 5). On the contrary, in AMC-treated calves, relative quantification raised for *bla*_TEM_, *strB*, *sul1*, *sul2*, and *intI1* genes at D6, decreased at D35, and remained mostly constant at D55. In particular, the *bla_TEM_* gene was four-fold more abundant in the AMC-treated group at D6 compared to D0 (*P* = 0.0155), whereas it underwent a four-fold decrease in the untreated group. The *bla*_TEM_ abundance decreased at D35 for the AMC-treated calves and maintained similar levels at D55 similar to the levels identified in untreated calves. Among ESBL-encoding genes, only three calves from the untreated group were positive for *bla*_CTX-M-1_ at D0. At D6, nine calves were tested positive; however, they were different individuals than those detected positive at D0. A similar situation occurred at D35, hindering a proper comparison between AMC-treated and untreated calves. Quantification of the *bla*_CTX-M-1_ gene was similar in AMC-treated and untreated calves at D6 (Table S5).

Considering that some calves received fluoroquinolones between D0 and D6 (Fig. 1), mean values of relative quantification of *qnrB1* and *qnrS* genes from AMC-treated and untreated calves were analyzed together. The relative quantification of both genes increased between D0 and D6 from 9.895 × 10^−8^ to 4.159 × 10^−6^ number of copies for the *qnrB1* gene and from 1.224 × 10^−4^ to 1.315 × 10^−1^ for the *qnrS* gene.

## DISCUSSION

Our study proved that a five-day AMC treatment by intramuscular injection had an impact on the microbiota of three weeks aged calves, at different levels. Calves enrolled for this study were exposed to other antibiotics, which could have had an impact on the calf’s microbiota composition, as well. Those from farm A received flumequine and doxycycline to treat intestinal and respiratory conditions, respectively. Calves from farm B received further antibiotics at different times, like oxytetracycline and neomycin for intestinal disorders between D0 and D6, and tilmicosin and doxycycline between D6 and D35 to treat respiratory conditions. To the best of our knowledge, longitudinal studies analyzing the effect of flumequine on calf’s microbiota do not exist. However, oxytetracycline effect has been investigated in previous studies demonstrating to impact bovine microbiota composition (25, 31, 32) and to increase tetracycline resistance genes (25, 31). Neomycin had an impact on bovine gastrointestinal metabolism along with a decrease of abundance of *Lactobacillus* and *Bifidobacterium* (33). The effect of these antibiotics on the microbiota of calves of this study cannot be analyzed because all calves received these antibiotics, and there was not an untreated control group. This is a limitation of this study since exposure of calves to other antibiotics could be a confounding effect; however, this is unfortunately inherent to on-field studies. Nevertheless, AMC treatment was the only differentiating factor between AMC-treated calves and the untreated control group so that differences that will be observed in the microbiota composition can ultimately be attributed to the AMC treatment.

Upon arrival at the farms, gut microbiota exhibited diversity among veal calves, likely due to their birth in various farms, which offered different nutrition and hygienic conditions, but also due to the stress induced by their transport to the fattening center (15, 23). The gut microbiota was dominated by the Bacillota phylum as expected for pre-weaned calves at this developmental stage (34, 35). Although the relative abundance of Pseudomonadota is expected to decrease as calves age (36), the microbiota of the treated veal calves was transiently dominated by this phylum following AMC treatment. At the family level, Enterococcaceae and Enterobacteriaceae strongly increased in AMC-treated calves. Consistent with this observation, *Escherichia* and *Shigella* genera were significantly more abundant in AMC-treated calves when compared to the untreated group. An increase of the *Escherichia* genus or Enterobacteriaceae family relative abundance was also observed following AMC treatment in dogs (11), cats (37), and healthy adult humans (38). In parallel, a decrease of the Lachnospiraceae family was noted in AMC-treated calves, mirroring findings in AMC-treated dogs (11) and humans (38). The occurrence of diarrhea has been found to be negatively correlated with the presence of Lachnospiraceae in calves’ gut (39, 40). Furthermore, members of the Lachnospiraceae family are butyrate and other short-chain fatty acids (SCFA) producers, which play a protective role against inflammation (41). In calves of this study, differences in microbiota composition were attributed to factors beyond diarrhea (since AMC-treated calves did not show specific symptoms compared to non-treated ones), including the sampling time and the AMC treatment. The evolution in the composition of the calves’ microbiota over time was in line with earlier findings (5, 42, 43). At D0, *Blautia*, *Faecalibacterium,* and *Clostridium* dominated calves’ microbiota, with some exceptions, similar to the study of Massot et al. (23) that reported these genera among the most abundant in 21 days old calves. The relative abundance of these genera increased throughout the study period with no significant differences between the AMC-treated and untreated calves at D35 and D55.

The Bray-Curtis analysis highlighted that microbiota composition of AMC-treated calves at D6 diverged from that observed at D0, D35, and D55 although three calves ranked differently. Breed, age, or any other metadata could not explain such differences. Nonetheless, all calves, including AMC untreated ones, received other antibiotics (flumequine in farm A, oxytetracycline and neomycin in farm B). At D6, an overall reduction of alpha-diversity was observed in both AMC-treated and untreated calves. The Shannon index increased following D6 and remained unaffected by the exposure to doxycycline and tilmicosin between D6 and D35. However, determining the impact of this administration on the microbiota of calves remains challenging. First, there was no doxycycline and tilmicosin untreated control group, as all calves in the farms were exposed to this treatment. Second, the timing of the stool sampling may have been too distant from the administration of doxycycline and tilmicosin between D6 and D35. Prior studies have indicated a transient effect of oxytetracycline on the microbiota composition and limitedly to the therapy duration (25). It is plausible that doxycycline may also exert a transient effect, considering that its spectrum of activity closely resembles that of oxytetracycline. The exposure to these antibiotics did not significantly affect the relative quantification of the *tet(A)* gene. On the contrary, the relative quantification of *qnrS* and *qnrB* genes, which confer quinolone resistance, increased after flumequine exposure. Similarly, the *bla*_TEM_ gene was significantly more abundant in AMC-treated calves than untreated ones and at D6 compared to D0. The relative quantification of *intI1*, indicator of multidrug-resistance, and the *sul1* gene, component of the 3′ conserved region of class one integrons (44), underwent a similar dynamic to that of the *bla*_TEM_ gene, suggesting co-selection of multidrug-resistant bacteria in calves’ gut by the AMC treatment. Similar dynamics of *bla*_TEM_ and *intI1* gene abundance were observed also in amoxicillin-treated calves, where an increase of *tet(A*) and *strA/B* genes was observed (26).

Detection of genes encoding ESBL enzymes, like *bla*_CTX-M-1_ and *bla*_CTX-M-9_ variants, was observed in a few calves by qPCR. The cultivation of stools on plates selective for ESC-R *E. coli* demonstrated higher sensitivity although in few cases qPCR amplified *bla*_CTX-M-1_ gene from stools that were negative by cultivation. Lack of amplification in calves positive by cultivation could have been due to the presence of qPCR inhibitors. Upon arrival, the rate of calves positive for ESC-R *E. coli* differed in the two farms (30% in farm A and 10% in farm B), but the number of screened calves also varied at D0 with only 10 calves available in farm A and 61 in farm B. Each farm displayed a specific dynamic of ESC-R *E. coli* occurrence. In farm A, ESC-R *E. coli* disappeared at D6 and D35. However, at D55, the prevalence of these bacteria increased, concerning 56% of resident calves and 100% of calves that had received AMC. In farm B, there was an increase in ESC-R *E. coli* at D6, followed by a gradual decrease by D55. The ESC-R *E. coli* increase observed in AMC-treated calves was not significantly different from the occurrence in untreated calves. A comparable finding was noted in dogs, indicating that the prevalence of ESC-R *E. coli* remained unchanged following AMC treatment (11). Other beta-lactams, such as cephalexin (45), ceftiofur, and cefquinome have demonstrated a clearer selective power on ESC-R *E. coli* than AMC (46, 47). Besides, selection of ESC-R *Enterobacter* spp. has been observed in humans treated by AMC (10). The global dynamic observed in farm B was in line with what has been reported in the Netherlands (48) and in France (20) where a temporal decline of ESC-R *E. coli* was observed. Similarly, a study carried out in Germany examining a broader resistance rate revealed a decrease in antibiotic-resistant bacteria as calves aged (49). However, each farm can display a specific dynamic scheme, as already reported (17) and as observed here for farm A, which can largely diverge from the general trend.

The molecular characterization of the 104 ESC-R *E. coli*, collected from 76 screened calves across all sampling times, revealed the presence of common molecular mechanisms underlining ESC-R. Seventeen isolates exhibited mutations within the promoter region of the intrinsic *ampC* gene. Only isolates with the C > T mutation at position –42 exhibited resistance to cefoxitin, suggesting the expression of the *ampC* gene (50). These mutants belonged to ST88. Mutations in the *ampC* promoter region have been previously documented in ST88 or ST23 in cattle (51). More frequently, ESC-R *E. coli* carried *bla*_CTX-M-group1_ (*n* = 69) and its variants *bla*_CTX-M-15_ (*n* = 6), *bla*_CTX-M-55_ (*n* = 14), and *bla*_CTX-M-32_ (*n* = 1). The *bla*_CTX-M-14_ and *bla*_SHV-12_ were found in five isolates each. The genetic determinants carrying the *bla*_ESBL_ genes were not characterized although an *in silico* analysis predicted a plasmid location for most of *bla*_CTX-M-1_ genes and *bla*_CTX-M-55_. Localization of the other *bla*_CTX-M_ variants and *bla*_SHV-12_ was predicted mostly on the chromosome. However, our study aimed to assess the global impact of AMC on bacterial population and resistance genes abundance, rather than deciphering the dissemination routes of resistance genes.

The most prevalent STs were ST2325 (*n* = 35), ST744 (*n* = 22), and ST34 (*n* = 17). These STs have been previously reported from the stools of various animals (52–56). The ST744 is a worldwide-diffused clone hosting also carbapenem-resistant isolates (57). However, the core-genome phylogenetic analysis suggests a lack of overlap between isolates of animal and human origins (53, 58). *E. coli* isolates belonging to ST2325 from different sources share high genomic similarity, suggesting inter-host circulation of this lineage (59). Multidrug-resistant *E. coli* isolates belonging to ST34 have been isolated also from healthy humans (60) and infected pediatric patients (61), suggesting that this lineage deserves further investigation to understand its One Health relevance. The ST2325 ESC-R *E. coli* were exclusively collected from farm B across various sampling times and were endemic in farm B being present in calves located in distant zones of the farm and even on a feeder and a bottle’s nipple. The ST34 ESC-R *E. coli* was also exclusively detected in farm B and was a successful clone, occurring in multiple calves hosted in distant zones and a bottle’s nipple. ESC-R *E. coli* ST744 were collected from calves in both farms. The isolate collected at D0 from farm A was distinct from those isolated at D35 and D55. In farm B, the ST744 isolates were identified solely at D35 from calves inhabiting zone 4, indicating potential dissemination among calves through direct contact. Further than calf-to-calf contact, other factors can contribute to bacterial dissemination and persistence in the farm environment, including recycling of clones through boots of farm operators contaminated by feces (62), insects (63), and even the air (64).

### Conclusion

The treatment with AMC affected the gut microbiota of calves, demonstrating effects even after a single therapy. Exposure of the gut to AMC lead to a reduction in bacterial diversity. The increase of Enterobacteriaceae and the reduction of Lachnospiraceae, known for producing SCFAs, are characteristic effects of AMC on the microbiota across various mammals. Nevertheless, the production of beneficial SCFAs could be compensated by other bacterial families. To comprehend the influence of AMC on the production of these beneficial compounds for host health, alternative approaches are required, such as those using shotgun metagenomics. AMC selected antibiotic resistance genes that are relevant in veterinary contexts and serve as indicators of multidrug resistance. The effect of AMC on the selection of ESC-R *E. coli* is less clear even though the increase of Enterobacterales might lead to clonal expansion of ESC-R bacteria within the calves’ gut. Calves enrolled in this study all received other antibiotics than AMC, an issue that is proper to on-field investigations and might represent a confounding factor. However, these were group treatments so that the potential effects of these antibiotics on the microbiota are expected to be the same in AMC-treated and non-treated calves. Since AMC is the only antibiotic that was exclusively administered to the AMC-treated group, we assumed that differences in the microbiota between the two groups can ultimately be attributed to the AMC treatment. Dissemination of ESC-R *E. coli* then likely occurred through direct contact among calves. Certain clones exhibited extensive spatial distribution, indicating the potential role of mobile vectors, such as footwear of farm operators or feeding facilities. To limit dissemination of antibiotic-resistant bacteria of One Health relevance, like ESC-R *E. coli*, improving hygienic conditions in farms is crucial. In all, our results further comfort the EMA categorization of AMC as a class C antibiotic that should be used only when the first line options are not any more effective.

## Data Availability

Reads and assemblies of 16S V1–V9 region and *E. coli* isolates sequencing are available from the BioProjects PRJNA1231232 and PRJNA1242555. R scripts, packages, and versions used are available from Gitlab (https://github.com/uavb/2024_meta16s_minion_Veaux.git).

## SUPPLEMENTAL MATERIAL

Figure S1 spectrum.01316-25-s0001.pdf Maps and location of calves in farms.

Figure S2 spectrum.01316-25-s0002.pdf Phylogenetic trees from SNP analysis.

Supplemental text spectrum.01316-25-s0003.docx Additional details of bioinformatic analysis of 16S rRNA V1-V9 region sequencing and *E. coli* genome sequencing.

Table S1 spectrum.01316-25-s0004.xlsx Features of enrolled calves and occurrence of extended-spectrum cephalosporin-resistant *E. coli*.

Table S2 spectrum.01316-25-s0005.xlsx Log2-fold change of bacterial relative abundance in AMC-treated and untreated calves and at different sampling times.

Table S3 spectrum.01316-25-s0006.xlsx Features of ESC-R *E. coli* isolates.

Table S4 spectrum.01316-25-s0007.xlsx SNP matrix of ESC-R *E. coli* isolates belonging to the same sequence type.

Table S5 spectrum.01316-25-s0008.xlsx Ratio of antibiotic resistance genes (number of copies) and 16S rRNA encoding gene.

ASM does not own the copyrights to Supplemental Material that may be linked to, or accessed through, an article. The authors have granted ASM a non-exclusive, world-wide license to publish the Supplemental Material files. Please contact the corresponding author directly for reuse.
