# Supplementary material for: Changes in fecal microbiota after therapeutic exposure to amoxicillin-clavulanic acid in veal calves receiving multiple antibiotics
Source: Microbiol Spectr. 2025 Nov 10;13(12):e01316-25. doi: 10.1128/spectrum.01316-25 (PMC12671132; doi:10.1128/spectrum.01316-25)
Supplement: Figure S1 — Maps and location of calves in farms. [file spectrum.01316-25-s0001.pdf]

Farm A map at D0 and D6

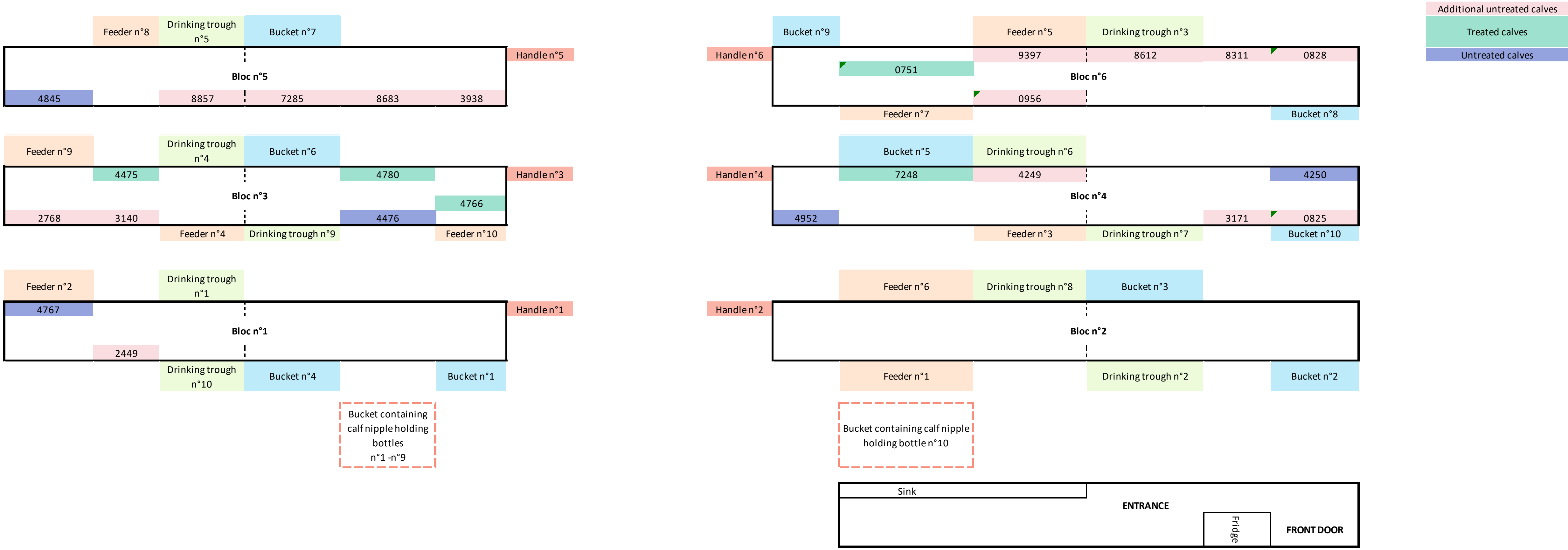

Farm A map at D35 and D55

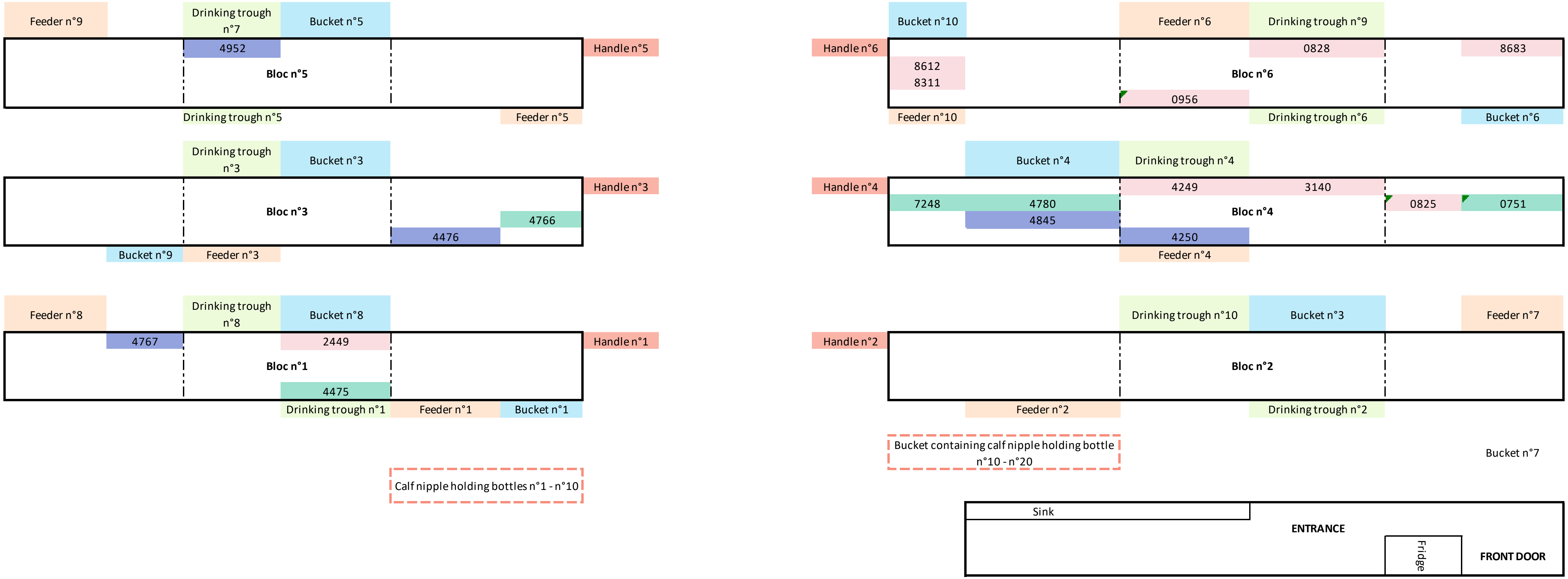

Farm B map at D0 and D6

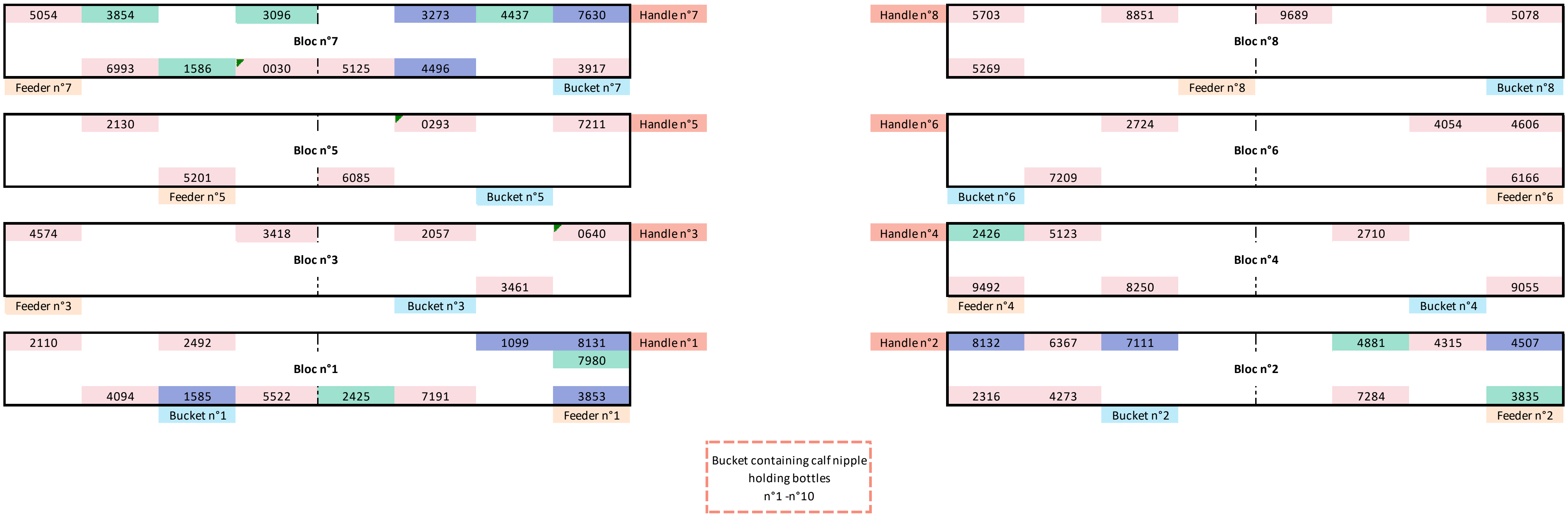

Farm B map at D35 and D55

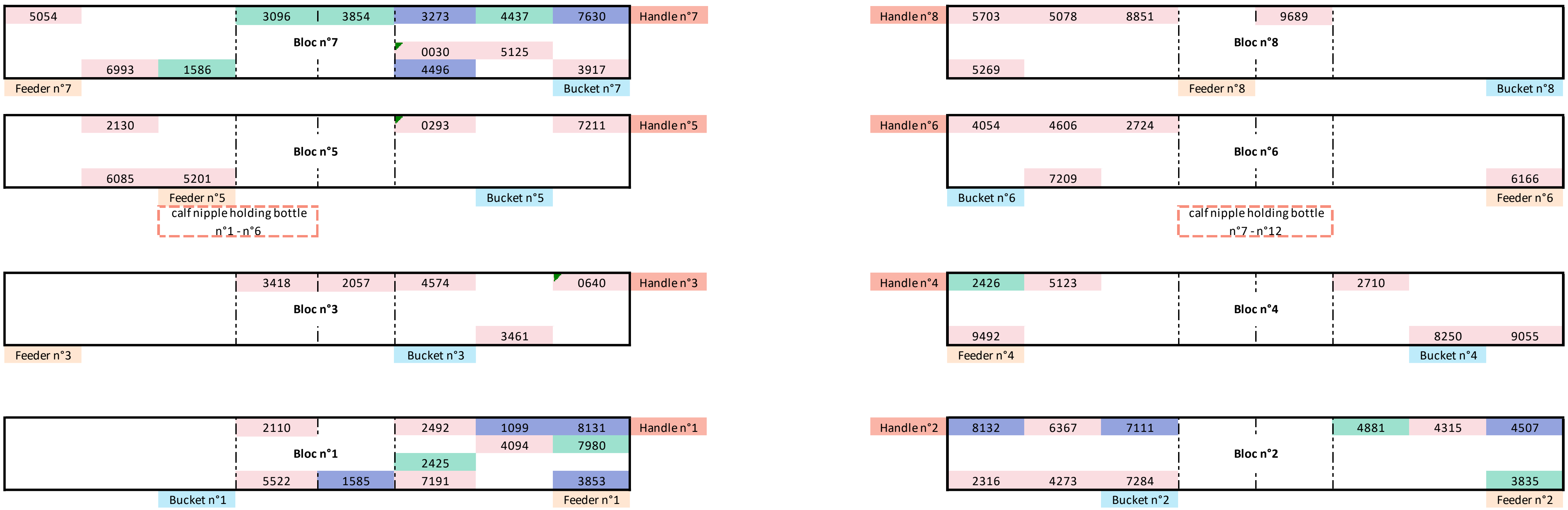

Figure S1. Graphical representation of farms regions and groupment of calves.
