# Supplementary material for: Changes in fecal microbiota after therapeutic exposure to amoxicillin-clavulanic acid in veal calves receiving multiple antibiotics
Source: Microbiol Spectr. 2025 Nov 10;13(12):e01316-25. doi: 10.1128/spectrum.01316-25 (PMC12671132; doi:10.1128/spectrum.01316-25)
Supplement: Figure S2 — Phylogenetic trees from SNP analysis. [file spectrum.01316-25-s0002.pdf]

Figure S2. Phylogenic trees from SNP analysis (Table S3). Antibiotic resistance genes and plasmid content were added, as well as the information of the location of calves within the farms. Gray shadowing of isolates labels highlights isolates from farm A. Legend for the time samples is found in the Figure S1A.

A. ST2325

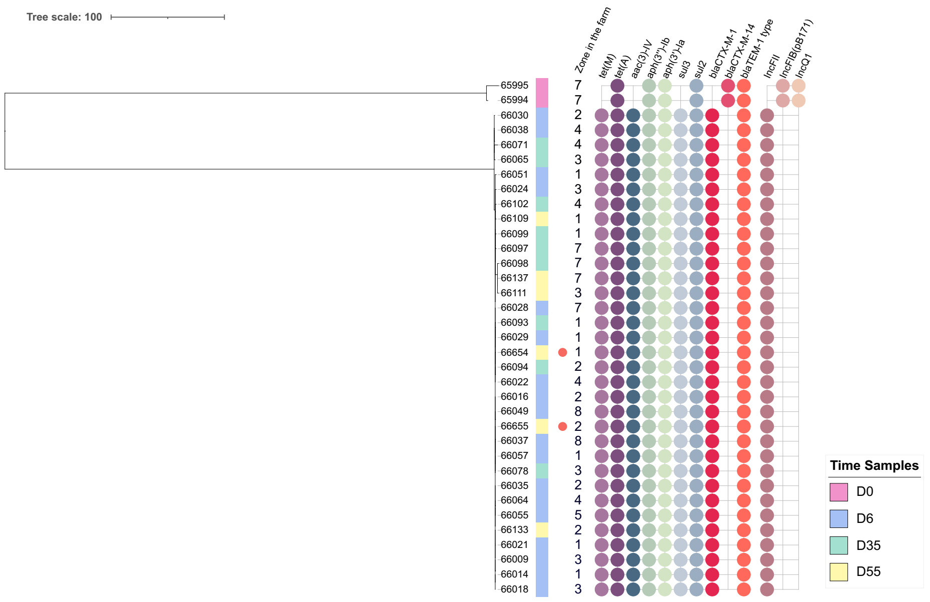

B. ST744

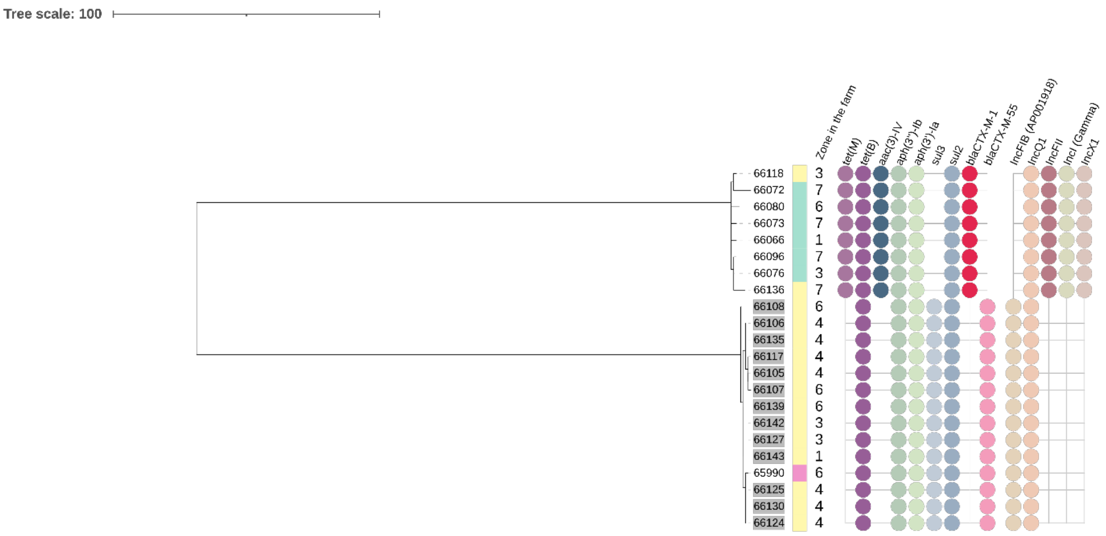

C. ST34

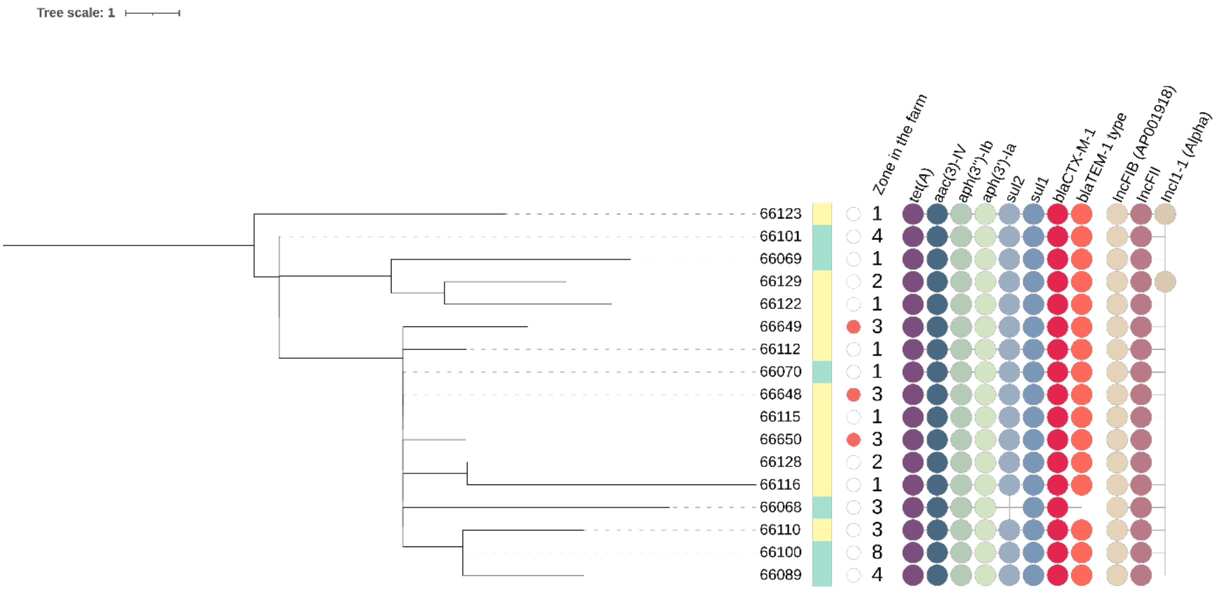
Tree scale: 1
Zone in the farm
tet(A)
aac(3)-IV
aph(3'')-Ib
aph(3')-Ia
sul2
sul1
blaCTX-M-1
blaTEM-1 type
IncFIB (AP001918)
IncFII
IncI1-1 (Alpha)
66123 1
66101 4
66069 1
66129 2
66122 1
66649 3
66112 1
66070 1
66648 3
66115 1
66650 3
66128 2
66116 1
66068 3
66110 3
66100 8
66089 4

D. ST58

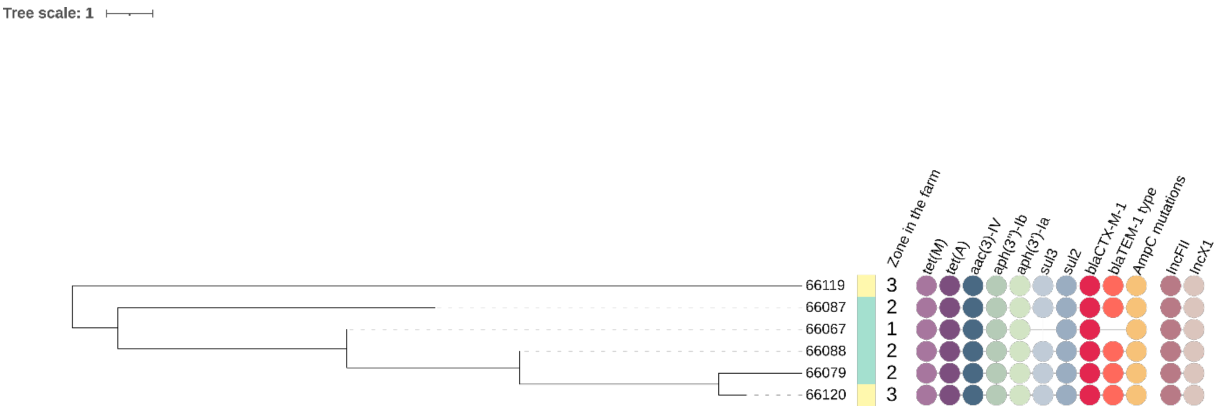
Tree scale: 1
Zone in the farm
tet(M)
tet(A)
aac(3)-IV
aph(3'')-Ib
aph(3')-Ia
sul3
sul2
blaCTX-M-1
blaTEM-1 type
AmpC mutations
IncFII
IncX1
66119 3
66087 2
66067 1
66088 2
66079 2
66120 3

E. ST10

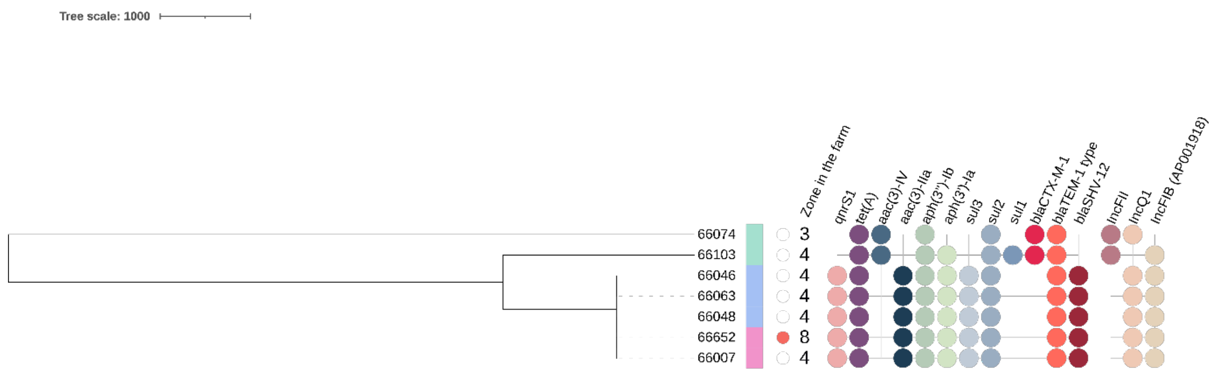

Tree scale: 1000
Zone in the farm
qnrS1
tet(A)
aac(3)-IV
aac(3)-IIa
aph(3'')-Ib
aph(3')-Ia
sul3
sul2
sul1
blaCTX-M-1
blaTEM-1 type
blaSHV-12
IncFII
IncQ1
IncFIB (AP001918)
66074
66103
66046
66063
66048
66652
66007
3
4
4
4
4
8
4
