# Supplementary material for: Changes in fecal microbiota after therapeutic exposure to amoxicillin-clavulanic acid in veal calves receiving multiple antibiotics
Source: Microbiol Spectr. 2025 Nov 10;13(12):e01316-25. doi: 10.1128/spectrum.01316-25 (PMC12671132; doi:10.1128/spectrum.01316-25)
Supplement: Supplemental text — Additional details of bioinformatic analysis of 16S rRNA V1-V9 region sequencing and E. coli genome sequencing. [file spectrum.01316-25-s0003.docx]

**Supplementary material 1**

**Supplementary methods for 16S rRNA gene sequencing and data analysis**

Cycling steps consisted in initial denaturation (1’ at 95°C); followed by 25 cycles including denaturation (20” at 95°C), annealing (30” at 55°C), extension (2’ at 65°C) and a final extension step (5’ at 65°C). The PCR products were dosed using the Qubit 4 fluorometer and equal amounts of amplicons per sample were used for libraries preparation using the Native Barcoding Kit 24 V14 (SQK-16S114.24) and following the manufacturer’s instructions (Oxford Nanopore Technologies, UK). Sequencing was performed on an Mk1B MinION sequencer using an R10.4.1 flow cell (FLO-MIN114). Basecalling was performed with Dorado (v.0.4.2).

Bioinformatic analyses were conducted using the epi2me/wf-metagenomics (v.2.10.0) pipeline (Oxford Nanopore Technologies, UK) for evaluating the number of obtained reads and their quality. The taxonomic assignation that was obtained using kraken2 (v2.1.3) and Standard-8 database (K2_standard_08gb_.20231009 and new_taxdump_11062024.tar.gz). Quality analysis was performed using NanoFilt, with the parameter –q equal to 10, to maintain only quality over 10, and length equal to 1000, to keep only reads’ length over 1000 (1).

Further analyses were conducted using R (v.4.2.2). A phyloseq object was obtained with the phyloseq package (v1.42.0). A Total Sum Scaling (TSSc) normalization was applied to the phyloseq object to create the relative abundance plots for various taxonomic levels (phylum, family, genus and species). The phyloseq object was converted using the phyloseq_to_deseq2 function (DESeq2, v.1.38.3). Afterwards, the data underwent normalization by applying the “EstimateSizeFactors” function, employing “poscount” as the fit type, which considers features with zero counts (2). Only log2fold changes with an adjusted p-value below 0.01 were reported. On the original phyloseq object, the microbiomeutilities R package (1.00.17) was used to estimate alpha diversity, based on the Shannon diversity index, and Wilcoxon tests were performed to estimate differences between AMC-treated and untreated veal calves and during sampling times (T0, T1, T2, and T3). Bray-Curtis dissimilarity analysis was represented on a Non-Metric Multi-Dimensional Scaling (NMDS) plot. PERMANOVA was performed on the differential matrix which was constructed at the genus level. To identify differentially abundant taxa between AMC-treated and untreated calves and time samples, linear mixed models with mixed effects were built. Models were built for each bacterial taxa, as fixed effects the relative abundance and ‘Time’, ‘Condition and their interaction, and as random effects in the format (~1+Time|Calves), that accounts for variability within calves. The normality of residuals and the heteroscedasticity were both checked for each model. Heteroscedasticity was modeled in the format ‘weights = varIdent (~1|Condition).

**Supplementary methods for Whole Genome Sequencing of *E. coli***

After sequencing, reads were trimmed and de novo assembled using Shovill v1.0.4. The quality of assemblies was assessed using Quast (v5.2.0) (3), Kraken2 (v2.1.2) (4) and Busco (v5.7.1) (5). The presence of resistance genes was investigated using ResFinder (v4.6.0) (6,7). The presence of plasmids was also investigated using PlasmidFinder (v2.2.0) (8). The presence of antibiotic resistance on plasmidic or chromosomic regions was analyzed using mlplasmids software (9).

Sequence types (STs) were assigned according to the Achtman scheme (MLST v2.23.0). A core-genome (2513 genes) MLST (cgMLST) was performed using pyMLST (v2.1.5) (10). The phylogenetic tree was generated using GrapeTree (v2.1) with neighbor joining method and was represented using iTOL v7 (11).

For isolates belonging to the same ST, an analysis of SNPs in the pangenome using PPanGGOLiN v2.1.0 was conducted using Gubbins (v3.3.5), avoiding recombination regions. Obtained trees were represented using tree builder RaxmlHPC avx2 (v8.2.12), using the model GTRGAMMA (12–14).

**References**

1. De Coster W, D’Hert S, Schultz DT, Cruts M, Van Broeckhoven C. NanoPack: visualizing and processing long-read sequencing data. Bioinformatics. 2018 Aug 1;34(15):2666–9.

2. Nearing JT, Douglas GM, Hayes MG, MacDonald J, Desai DK, Allward N, et al. Microbiome differential abundance methods produce different results across 38 datasets. Nat Commun. 2022 Jan 17;13(1):342.

3. Mikheenko A, Prjibelski A, Saveliev V, Antipov D, Gurevich A. Versatile genome assembly evaluation with QUAST-LG. Bioinformatics. 2018 Jul 1;34(13):i142–50.

4. Wood DE, Lu J, Langmead B. Improved metagenomic analysis with Kraken 2. Genome Biol. 2019 Nov 28;20(1):257.

5. Manni M, Berkeley MR, Seppey M, Simão FA, Zdobnov EM. BUSCO Update: Novel and Streamlined Workflows along with Broader and Deeper Phylogenetic Coverage for Scoring of Eukaryotic, Prokaryotic, and Viral Genomes. Mol Biol Evol. 2021 Oct 1;38(10):4647–54.

6. Bortolaia V, Kaas RS, Ruppe E, Roberts MC, Schwarz S, Cattoir V, et al. ResFinder 4.0 for predictions of phenotypes from genotypes. J Antimicrob Chemother. 2020 Dec 1;75(12):3491–500.

7. Camacho C, Coulouris G, Avagyan V, Ma N, Papadopoulos J, Bealer K, et al. BLAST+: architecture and applications. BMC Bioinformatics. 2009 Dec 15;10(1):421.

8. Carattoli A, Zankari E, García-Fernández A, Voldby Larsen M, Lund O, Villa L, et al. In silico detection and typing of plasmids using PlasmidFinder and plasmid multilocus sequence typing. Antimicrob Agents Chemother. 2014 Jul;58(7):3895–903.

9. Arredondo-Alonso S, Rogers MRC, Braat JC, Verschuuren TD, Top J, Corander J, et al. mlplasmids: a user-friendly tool to predict plasmid- and chromosome-derived sequences for single species. Microb Genomics. 2018;

10. Biguenet A, Bordy A, Atchon A, Hocquet D, Valot B. Introduction and benchmarking of pyMLST: open-source software for assessing bacterial clonality using core genome MLST. Microb Genomics. 2023;9(11):001126.

11. Letunic I, Bork P. Interactive Tree of Life (iTOL) v6: recent updates to the phylogenetic tree display and annotation tool. Nucleic Acids Res. 2024 Jul 5;52(W1):W78–82.

12. Gautreau G, Bazin A, Gachet M, Planel R, Burlot L, Dubois M, et al. PPanGGOLiN: Depicting microbial diversity via a partitioned pangenome graph. PLoS Comput Biol. 2020 Mar 19;16(3):e1007732.

13. Croucher NJ, Page AJ, Connor TR, Delaney AJ, Keane JA, Bentley SD, et al. Rapid phylogenetic analysis of large samples of recombinant bacterial whole genome sequences using Gubbins. Nucleic Acids Res. 2015 Feb 18;43(3):e15.

14. Stamatakis A. RAxML version 8: a tool for phylogenetic analysis and post-analysis of large phylogenies. Bioinformatics. 2014 May 1;30(9):1312–3.
